# The smell of fear: innate threat of 2,5-dihydro-2,4,5-trimethylthiazoline, a single molecule component of a predator odor

**DOI:** 10.3389/fnins.2015.00292

**Published:** 2015-08-25

**Authors:** Jeffrey B. Rosen, Arun Asok, Trisha Chakraborty

**Affiliations:** Department of Psychological and Brain Sciences, University of DelawareNewark, DE, USA

**Keywords:** predator, odor, fear, 2,5-dihydro-2,4,5-trimethylthiazoline, TMT, olfaction

## Abstract

In the last several years, the importance of understanding what innate threat and fear is, in addition to learning of threat and fear, has become evident. Odors from predators are ecologically relevant stimuli used by prey animals as warnings for the presence of danger. Of importance, these odors are not necessarily noxious or painful, but they have innate threat-like properties. This review summarizes the progress made on the behavioral and neuroanatomical fundamentals of innate fear of the predator odor, 2,5-dihydro-2,4,5-trimethylthiazoline (TMT), a component of fox feces. TMT is one of several single molecule components of predator odors that have been isolated in the last several years. Isolation of these single molecules has allowed for rapid advances in delineating the behavioral constraints and selective neuroanatomical pathways of predator odor induced fear. In naïve mice and rats, TMT induces a number of fear and defensive behaviors, including robust freezing, indicating it is an innate threat stimulus. However, there are a number of behavioral constraints that we do not yet understand. Similarly, while some of the early olfactory sensory pathways for TMT-induced fear are being delineated, the pathways from olfactory systems to emotional and motor output regions are less well understood. This review will focus on what we know and what we still need to learn about the behavior and neuroanatomy of TMT-induced fear.

Understanding how environmental stimuli motivate and influence behavior is one of the fundamental questions in behavioral neuroscience. Over the last several decades there has been tremendous progress toward answering this question. One of the more successful endeavors has been the study of defensive behaviors produced by threatening, dangerous, and fearful stimuli in rodents (Davis et al., 2010; LeDoux, 2012). This has primarily been studied using associative fear conditioning paradigms whereby a neutral stimulus, typically a tone, light, or context, is paired with an aversive stimulus, usually a foot shock, to become a fear conditioned stimulus. Fear conditioning research has been extremely informative for understanding how threat (i.e., fear) is processed across different levels of analysis (e.g., behavior, neuroanatomy, neurochemistry, genetics). In particular, fear conditioning research has provided insight into how animals behaviorally adapt, anticipate, and respond to a vast number of aversive and threatening environmental stimuli and potentially threatening situations. Importantly, Pavlovian fear conditioning paradigms have been instrumental for delineating the neural circuits necessary for learning about threats and fears that route through the amygdala (these will be discussed later). These circuits and the cellular and molecular mechanisms within these circuits important for learning and expressing fear are continually being refined (Herry and Johansen, 2014; Do-Monte et al., 2015; Janak and Tye, 2015).

Although the ability to adapt, anticipate, and learn during situations of threat and danger is paramount for survival, another way to increase survival in the face of danger is to have hardwired “learning-independent” systems. That is, systems for assessing threatening stimuli having prepotent qualities, which drive defensive behavior without prior learning. Many animals, including humans, appear to have species-specific innate sensitivity to the threat (and appetitive) qualia of various stimuli. In humans, fears, and phobias may cluster into at least three spheres: heights, blood/injection/injury, and situational/animal (de Jongh et al., 2011). Many people have a fear of spiders or of snakes (Oosterink et al., 2009), which may be innate or have prepotency for quick learning (Ohman and Mineka, 2001; Poulton and Menzies, 2002; Rachman, 2002). Predators (e.g., cats, foxes, weasels) of mice and rats, and odors from these predators, appear to have these prepotent qualities and have therefore been used to study behavior to innate threat or fear in the laboratory (Dielenberg and McGregor, 2001; Rosen, 2004; Masini et al., 2006; Blanchard et al., 2013; Takahashi, 2014; Brennan and Keverne, 2015). The innate quality of these predator stimuli is implied by the fact that the stimuli produce robust defensive behaviors upon the first exposure in laboratory rodent strains that have not encountered these predators for generations. While live predators are more natural and present the full complement of multimodal threat stimuli, their complexity hinders studying the neural circuitry from selective sensory input to the defensive behavioral outputs in the laboratory. The use of predator odors has been beneficial to this end in that they have allowed for the precise examination of defensive behaviors produced by stimulating a single sensory modality. Although attempts have been made to gain better control of the sensory features stimulated by live predators (e.g., a robotic “predator” has been developed to simulate a moving and looming threat Choi and Kim, 2010), they are limited in that they provide an artificial threat and it is unclear how selective or innate the threat actually is. Predator odors, particularly single molecule components of these natural odors, have high sensory selectivity and have therefore become increasingly studied in neuroscience laboratories.

Whereas live predators are innate fear-inducers, and thus are simplified models of fear relative to conditioned fear because no learning is necessary, predator odors are even simpler models because the odor primarily limits the sensory systems involved to olfaction. Predator odors such as those derived from bodily secretions (e.g., urine and feces) are kairomones—semiochemicals emitted by an organism that benefits an organism of another species—and trigger the expression of innate fear behaviors in prey. However, single molecule components of natural odors derived from predator secretions are the simplest models used to induce fear because they reduce the sensory system to fewer receptors than natural, complex predator odors. Furthermore, these single molecule components (referred to as single molecule predator odors throughout the rest of the manuscript) should facilitate the discovery of precise neurobiological and neuroanatomical circuitries of fear (Stowers and Logan, 2010).

There are a number of recently identified single molecule predator odors that have been shown to induce innate fear and defensive behaviors in rodents and other animals. In particular, research has identified a class of single molecule olfactory signals, the major urinary proteins (MUPs), which are capable in and of themselves in driving defensive behaviors in rats and mice. For example, Feld4, a homolog of MUPs, was recently isolated from cat fur—a predator stimulus that has been used for several decades as a fear and anxiety-inducing stimulus (Papes et al., 2010). Feld4 in cat fur likely comes from cat saliva deposited on the fur during self-grooming behavior. It induces defensive behavior in mice (Papes et al., 2010). The same researchers also found that another MUP from rat urine (rMUP13) induced defensive behavior in mice. Both Feld4 and rMUP13 bind to the transient receptor potential cation channel, subfamily C, member 2 (TrpC2) receptor in the vomeronasal organ, but not the main olfactory bulb.

Another single molecule predator odor, 2-phenylethylamine, was isolated from a large number of carnivore urines, including bobcat, weasel, ferret and fox urine, and shown to induce defensive behavior in both rats and mice (Ferrero et al., 2011). The presence of 2-phenylethylamine was found to be more than 50 times higher in carnivore urines relative to non-carnivorous species. Importantly, a single odor receptor, trace amine-associated receptor 4 (TAAR4), was identified as the primary olfactory receptor responsible for elicitation of defensive behavior to 2-phenylethylamine (Ferrero et al., 2011). Another trace amine-associated receptor (TAAR13c) is also important for defensive behavior in zebrafish elicited by cadaverine and putrescine, two amines produced in decaying animal flesh (Hussain et al., 2013). Finally, single molecule kairomones isolated from fox feces (2,4,5-trimethylthiazoline, TMT), in addition to weasel and ferret anal secretions (2-propylthietane), were first isolated in the 1980s (Crump, 1980; Vernet-Maury et al., 1984) and have since been used as predator threats in their synthesized forms. Given that TMT was discovered before other single molecule predator odors, it has been the most extensively studied and yielded the most progress toward understanding the neuroanatomical circuits necessary for producing defensive behaviors after encountering these types of odors (for reviews see Rosen, 2004; Fendt et al., 2005a; Fendt and Endres, 2008; Takahashi, 2014; Brennan and Keverne, 2015). The remainder of this paper will focus on that progress and some aspects still left to be determined about the fear-inducing qualities of TMT, the neural systems and circuitry for innate fear, and defensive behaviors elicited by TMT.

## TMT as an unconditioned fear stimulus in rodents

TMT is a synthetic compound that was originally isolated from fox feces by Vernet-Maury in the 1980s (Vernet-Maury et al., 1984). Of a number of compounds isolated from fox feces, TMT appeared to have the most robust effect on avoidance, and therefore was touted and marketed as a natural rodent repellent (Vernet-Maury et al., 1984). TMT is a highly volatile, water insoluble molecule that contains sulfur. Sulfur is an element in carnivores' diet and digestion metabolites, and is important for the threat-inducing qualities of urine and feces (Nolte et al., 1994; Brechbühl et al., 2013), as a meatless diet depletes sulfur in coyote urine, and the urine produced from a meatless diet does not elicit defensive responses from mice (Nolte et al., 1994). Cat feces from a meatless diet also reduced mouse defensive behavior compared to feces from a carnivorous diet, although sulfur content was not tested in this study (Berton et al., 1998). Interestingly, mouse alarm pheromones, which signal conspecific danger, contain another thiazoline compound that is chemically similar to TMT, suggesting that both conspecific odor alarm signals and predator kairomones are transduced by the same olfactory receptors (Brechbühl et al., 2013). The highly volatile nature of TMT indicates that it might be a long distance olfactory threat signal to rodents. TMT is thought to be an unconditioned threatening stimulus because naïve, laboratory bred, and raised rats and mice display fear-like responses on their first exposure to TMT (Wallace and Rosen, 2000). Examining freezing as a fear response, TMT elicits freezing to the same levels that footshock-induced conditioned freezing does (Wallace and Rosen, 2001). Also similar to footshock-induced freezing, exposure to TMT can be titrated to produce low to high amounts of freezing (Wallace and Rosen, 2000; Endres et al., 2005), demonstrating a dose-response relationship between the number of TMT molecules and the amount of freezing or unconditioned fear. The unconditioned quality of TMT is also evident from the lack of habituation and sensitization to repeated exposures to TMT, in addition to the difficulty of TMT to support contextual fear conditioning (Wallace and Rosen, 2000; McGregor et al., 2002; Blanchard et al., 2003). Although habituation and cue and contextual conditioning have been demonstrated to cat fur odor (Blanchard et al., 2001; Dielenberg and McGregor, 2001; Takahashi et al., 2008), a lack of behavioral habituation, sensitization, and contextual fear conditioning has been extended to cat feces and ferret fur odor (Blanchard et al., 2003; Masini et al., 2006), suggesting different sources of predator odors have varied danger or threat inducing properties.

The difficulty of producing contextual conditioning to TMT does not suggest that TMT cannot support conditioning. Indeed, in test chambers that have more than one compartment, TMT can support conditioned responses. In a chamber where TMT is introduced in one compartment, rats will avoid that compartment when tested later without TMT present (Endres and Fendt, 2007). Similarly, in a chamber with a hide box, TMT exposure produces a small, but significant, increase in conditioned freezing the day following exposure (Rosen et al., 2008b). Conditioned changes in other behaviors were also found—increased time spent in the hide box, less exploration, and fewer contacts with the odor source (Rosen et al., 2008b).

## What we know about TMT-induced defensive behavior

TMT is the most widely studied single molecule predator odor because it produces robust, invariant levels of freezing behavior—a well-documented fear behavior in rats, mice, and even humans (Blanchard and Blanchard, 1969; Roelofs et al., 2010; Hagenaars et al., 2014). Considering that freezing is also the most studied behavior for fear conditioning experiments, unconditioned freezing to TMT provides support for the notion that a fear response is due to the odor's threat or fear inducing properties instead of other properties, such as its unpleasant, even acrid, qualities that elicit avoidance (Fendt and Endres, 2008; Ayers et al., 2013). TMT also elicits or reduces other behaviors associated with threat, such as eliciting flat-back stretching and decreasing exploration and rearing—behaviors that have been documented to be defensive in rats and mice in response to a cat and cat fur odor (Fendt and Endres, 2008; Rosen et al., 2008b).

Interestingly, other single molecule predator odors have not been shown (or not been tested) to elicit freezing, but have been shown to induce avoidance (Ferrero et al., 2011). We think it is important to demonstrate that a predator odor elicits well-documented fear or defensive behaviors as well as avoidance because avoidance can be a result of the unpleasant or noxious qualities of the odor rather than its fear-inducing properties (Wallace and Rosen, 2000; Fendt and Endres, 2008). Odors not associated with predators, like butyric acid and caproic acid, which have noxious acrid qualities, have been shown to induce avoidance, similar to many predator odors, but not induce significant freezing (Wallace and Rosen, 2000). Measuring the elicitation of documented defensive and fear behaviors in addition to avoidance is critical for the study of innate predator odors.

We also know that TMT doesn't readily support context conditioning (Wallace and Rosen, 2000; Blanchard et al., 2003), and this differs from cat fur odor, which does serve as an unconditioned stimulus for context and auditory conditioning in single chamber tests (Blanchard et al., 2001; Dielenberg and McGregor, 2001; Takahashi et al., 2008). However, in two-compartment test chambers TMT does support context conditioning of a number of defensive and avoidance behaviors (Endres and Fendt, 2007; Rosen et al., 2008b), suggesting that although TMT induces robust freezing in a simple single chamber, it needs some environmental complexity to support contextual fear conditioning.

## What we still need to learn about TMT-induced defensive behavior

This leads us to ask what we do not know about TMT induced behavior. Since TMT-induced conditioning is so circumscribed, a major question is, what are the environmental constraints of TMT supported conditioning? Size of test chamber, lighting conditions, familiarity, and prior safe experience of the environment modulate innate TMT-induced fear behavior (Morrow et al., 2002; Rosen et al., 2008b; Nikaido and Nakashima, 2009; Knox et al., 2012). Why are two (or more) compartment chambers necessary for supporting context-TMT conditioning? Furthermore, what types of neutral stimuli can be conditioned to TMT? An interesting study demonstrated that TMT supports flavor conditioned avoidance (Myers and Rinaman, 2005). Mice learned to avoid drinking flavor-infused water (combined taste and odor stimuli, e.g., almond or vanilla extract in water) that was previously paired with TMT compared to non-TMT paired flavors. This was selective for TMT, as avoidance did not occur when flavored-water was paired with exposure to banana extract, a non-predator odor. This study, in addition to studies needing complex test chambers for TMT-induced context conditioning (Endres and Fendt, 2007; Rosen et al., 2008b), suggests that more naturalistic stimuli or environments may produce more robust conditioning than context.

TMT, while not readily supporting contextual fear conditioning, might produce a generalized sensitization-like effect. Following exposure to TMT, adult mice were found to have enhanced acoustic startle responses, which lasted 3 days or more (Hebb et al., 2003). Rats and mice exposed to TMT also showed increased anxious behavior (less time spent in the open arms) on the elevated plus maze, in an open field test, and in a light-dark test lasting more than 3 days following TMT exposure (Hebb et al., 2002, 2004; Fendt et al., 2005a). This is similar to the well documented long-lasting anxiogenic effects of cat odors (Cohen et al., 2012). Further study of the generalized sensitization-like effects of TMT would be quite important for translational models of anxieties, phobias, and posttraumatic stress disorder (Rosen et al., 2008b).

However, another study did not find sensitization to TMT-induced freezing during chronic exposure to high levels of corticosterone (Rosen et al., 2008a). TMT induces corticosterone secretion (Day et al., 2004), which is known to enhance anxious behavior and fear conditioned freezing (Schulkin et al., 2005; Roozendaal et al., 2009), so it is difficult to reconcile the increase in prolonged sensitization following TMT on startle and the elevated-plus maze, and the lack of an effect on TMT-induced freezing with chronic corticosterone. Possibly, increased corticosterone associated with TMT needs an environmental context to produce long lasting anxiogenic effects. Or, increased corticosterone might be involved in potentiating defensive responses to other situations (i.e., generalized sensitization) after TMT exposure, like startle, elevated plus maze, and the other situations mentioned above. Clearly, there are still avenues and interesting features to discover about the defensive and fear behavior associated with TMT and TMT-induced physiological responses.

An additional question stems from the differences in the ability of TMT to induce fear responses in different strains of rats and mice. Sprague–Dawley and Long–Evans strains of rats display robust freezing to TMT, but Wistar rats do not (Rosen et al., 2006; Staples and McGregor, 2006). Similarly, CD-1 mice do not seem to respond to TMT with freezing behavior, but C57BL/6J mice do (Fortes-Marco et al., 2013). However, another lab has shown increases in TMT-induced freezing in CD-1 mice (Hebb et al., 2004), but didn't compare other mouse strains. Whether these strain differences are due to differences in the ability to perceive (smell) TMT would be important to answer, and would suggest that these differences are due to genetic variation in the olfactory receptor populations in these strains (Rosen et al., 2006). If olfactory receptor populations are different, then this can be exploited to discover the selective olfactory receptors responsible for TMT to induce fear responses.

What we know and still need to learn about TMT-induced fear behavior is summarized in Table 1.

**Table 1:** **TMT-induced defensive behavior**.

What we know about the TMT-induced defensive behavior TMT produces robust, invariant levels of freezing with repeated exposure - Lack of habituation - Lack of sensitization TMT can induce a large array of species-specific defensive behaviors - Size of text chamber, lighting conditions, familiarity, prior safe experience modulate freezing, and other defensive behaviors TMT can support context and flavor-avoidance conditioning of defensive behaviors
What we still need to learn about TMT-induced defensive behavior What types of stimuli can and cannot be conditioned to TMT? What are the environmental constraints of TMT-induced fear conditioning? TMT supports limited context conditioning, but good conditioning of flavor avoidance Why are more complex environments better for TMT-induced fear conditioning? How good is TMT as a model for anxiety disorders—phobias and posttraumatic stress disorder? Study on TMT-induced generalized sensitization, habituation, and extinction is needed.

## Neuroanatomy of TMT-induced fear

Studies investigating the neuroanatomy of predator odors have also made significant progress over the years. Most research has investigated cat and cat fur odor as innate predator stimuli (Blanchard et al., 2005; Gross and Canteras, 2012). The locus of attention has primarily been in the hypothalamus and amygdala using lesions, pharmacological inactivation, electrical stimulation, and markers of neuronal activation (for review see Gross and Canteras, 2012; Takahashi, 2014). Newer, molecular techniques (e.g., transgenic mice, optogenetics) are also being employed to identify the neuroanatomy and receptors for innate fear of predator odors (Root et al., 2014).

While some progress has been made on the “fear-associated” neuroanatomy of single molecule predator odors (that is, amygdala and hypothalamic circuitry), great progress has been made in elucidating the olfactory receptors and neuroanatomy for a variety of single molecule predator odors by starting at the sensory organ—the nasal epithelium. For example, individual olfactory receptors, or a class of olfactory receptors in the case of TMT, have been identified for Feld4, rMUP13, 2-phenylethylamine, and TMT. In mice, the transient receptor potential cation channel, subfamily C, member 2 (TrpC2) in the vomeronasal organ is critical for avoidance and risk assessment behavior to Feld4 and rMUP13 (Papes et al., 2010). In rats and mice the trace amine-associated receptor 4 (TAAR4) in the main olfactory bulb is important for avoidance behavior to 2-phenylethylamine (Ferrero et al., 2011; Dewan et al., 2013). In addition, sensory olfactory neurons with a class of olfactory receptors in zone II of the nasal epithelium, possibly with up to about 100 olfactory receptor genes called dorsal domain class II receptors (DII), synapse in the glomeruli of the dorsal portion of the main olfactory bulb. Most importantly this class of DII olfactory neurons are necessary for innate avoidance of TMT (Kobayakawa et al., 2007). This landmark study, which produced mutant mice lacking neurons with DII receptors and showed a lack of fear and anxious responses to TMT (Kobayakawa et al., 2007), has been a major impetus for the recent progress made in the neuroanatomical olfactory circuits necessary for processing single molecule predator odors and innate fear. It empirically demonstrated that a group of class II olfactory neurons in zone II of the nasal epithelium, which project to the dorsal part of the main olfactory bulb, was responsible for innate fear to TMT, a volatile predator odor.

A recent study also points to the Grueneberg ganglion as another olfactory system important for TMT-induced freezing (Brechbühl et al., 2013). The Grueneberg ganglion is an olfactory subsystem located at the tip of the nose close to the entry of the naris. It comprises neurons that are both sensitive to cold temperature and play an important role in the detection of alarm pheromones (Brechbühl et al., 2008, 2013). The olfactory receptors of the Grueneberg ganglion are particularly sensitive to methylated thiazolines, of which TMT is one. The neurons of the Grueneberg ganglion synapse in the dorsal main olfactory bulb (Brechbühl et al., 2013), possibly within the DII domain glomeruli (Kobayakawa et al., 2007). Transection of the axons of the Grueneberg ganglion cells blocks TMT-induced freezing (Brechbühl et al., 2013). Thus, it is likely that the elicitation of freezing by TMT is transduced through receptors in the nasal epithelium (Kobayakawa et al., 2007) and the Grueneberg ganglion (Brechbühl et al., 2013).

Although the studies just reviewed suggest that TMT's fear inducing qualities are transmitted to the brain via two olfactory systems, there is the possibility that the noxious property of TMT is transduced through the trigeminal nerve to drive the freezing, avoidance, and other behavioral effects of TMT (McGregor et al., 2002; Fendt and Endres, 2008; Galliot et al., 2012). To address this issue, our lab recently demonstrated that olfactory bulb ablation completely blocked the freezing response to TMT, whereas transection of the infraorbital and ethmoidal branches of the trigeminal nerve (eliminating noxious sensations from the nasal cavity, mouth region, and whiskers) had no effect of TMT-induced freezing (Ayers et al., 2013). This was complemented by a lack of an effect of olfactory bulb lesions on butyric acid-induced behavior, whereas the trigeminal transection reduced freezing to butyric acid (Ayers et al., 2013). These results indicate that TMT-elicited innate fear behavior is dependent on olfaction, but not its noxious properties. This effect of olfactory bulb ablation on TMT-induced freezing has recently been replicated (Taugher et al., 2015, this special issue).

**Figure 1:**
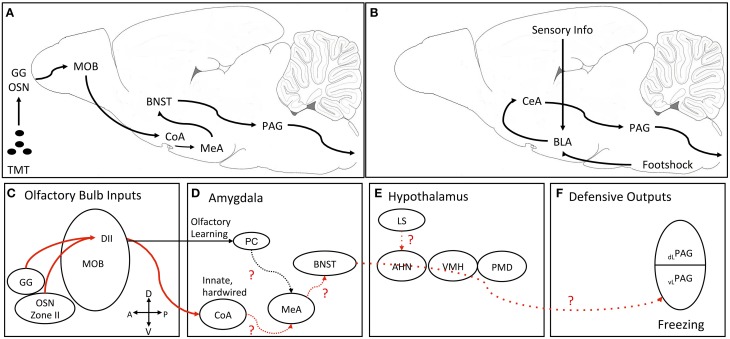
**Proposed neural processing pathway for TMT-induced freezing and pathway for fear conditioning**. **(A)** Simplified schematic of pathway from olfaction to freezing output. **(B)** Simplified schematic of the classic fear conditioning pathway where conditioned and unconditioned information impinges on the BLA and then gets sent to CeA which projects to the PAG. **(C–F)** Detailed schematic of the pathway for TMT-induced freezing. Solid red lines are empirically determined neural pathways for innate TMT-induced freezing. The dotted red lines are the proposed connections through nuclei known to be important for TMT-induced freezing. **(C)** TMT inputs from type II olfactory sensory neurons and the Grueneberg ganglion project to domain II glomeruli in the dorsal main olfactory bulb. These glomeruli project through two pathways. **(D)** One pathway from the mitral/tufted cells to the PC for TMT-related learning and another to the CoA for innate defensive processing. For innate TMT-induced freezing hypothesized that information is then relayed to the MeA and then serially to ventral parts of the BNST. **(E)** TMT information is then sent from the BNST to the PAG passing through the medial hypothalamic defensive circuit (AHN, VMH). An additional input for TMT-induced freezing is from the LS possibly projecting to the AHN. **(F)** Finally, it is hypothesized that TMT information from the BNST reaches the vlPAG to mediate defensive freezing. AHN, anterior hypothalamic nucleus; BLA, basolateral amygdala complex; BNST, bed nucleus of the stria terminalis; CeA, central nucleus of the amygdala; CoA, cortical nucleus of the amygdala; DI and DII, dorsal domain 1 and domain 2 glomeruli; GG, Grueneberg ganglion; LS, lateral septum; MeA, medial nucleus of the amygdala; MOB, main olfactory bulb; OSN, type II olfactory sensory neurons; dlPAG, dorsolateral periaqueductal gray; vlPAG, ventrolateral periaqueductal gray; PC, pyriform cortex; PMD, dorsal premammillary nucleus; VMH, ventromedial hypothalamus.

Advances in circuitry beyond the olfactory bulb have been made from the DII TMT-responsive neurons in the dorsal main olfactory bulb (Figures 1C–F). Optical imaging of the olfactory bulb found TMT-responsive glomeruli clustered in the posterior part of the DII domain (called cluster J; Matsumoto et al., 2010). The neurons from this posterior part of DII domain of the olfactory bulb have further been traced to synapse on mitral and tufted cells that project to the cortical amygdala (Miyamichi et al., 2010; Sosulski et al., 2011). Both studies speculated that olfactory circuits projecting to the cortical amygdala are responsible for the generation of innate behavior to olfactory stimuli. Functional studies using optogenetic methods demonstrated that TMT-induced innate avoidance and freezing were reduced by inhibition of mitral cells projections from the dorsal olfactory bulb to the cortical amygdala (Root et al., 2014). Optogenetic activation of anterior cortical amygdala neurons induced avoidance and freezing behaviors similar to those induced by TMT (Root et al., 2014). Together, these studies identify a functional two-synapse olfactory circuit for innate fear behavior to TMT. This circuit initiates fear behavior from class II olfactory sensory neurons in the nasal epithelium or Grueneberg ganglion cells, which synapse on mitral cells in cluster J glomeruli in the posterior dorsal olfactory bulb. From there, mitral cells synapse in the anterior cortical nucleus of the amygdala.

Where the cortical amygdala neurons synapse in the next leg of an innate olfactory fear circuit is not known at present (Figure 1D). However, using the immediate-early gene, c-fos, as an activation marker, TMT has been shown to induce activation in the medial part of the anterodorsal medial nucleus of the amygdala (Day et al., 2004) and muscimol inactivation of the medial nucleus of the amygdala significantly reduced TMT-induced freezing behavior (Müller and Fendt, 2006).

Once transduction of the TMT fear-inducing signal gets beyond the olfactory cortical regions and medial amygdala, it is not clear how the circuit proceeds to motor control regions important for fear and defensive behavior, such as the periaqueductal gray (PAG) to generate escape and freezing responses (Vianna and Brandão, 2003) (Figure 1E). Circuitry through three hypothalamic nuclei—the anterior hypothalamic nucleus, the dorsomedial division of the ventromedial hypothalamic nucleus and the dorsal premammillary nucleus—have been delineated for fear and defensive behavior induced by a live cat and cat fur odor, called the medial hypothalamic defensive circuit (Gross and Canteras, 2012). The dorsomedial division of the ventromedial hypothalamus (VMHdm) has also been shown to be activated by a number of single molecule predator odors that are transduced through various olfactory organs, including vomeronasal organ (Feld4), nasal epithelium (2-phenylethylamine), and Grueneberg ganglion (2-propylthietane) (Pérez-Gómez et al., 2015). Most interestingly, TMT did not activate c-fos in the VMHdm in this study and another study (Staples et al., 2008; Pérez-Gómez et al., 2015), but see (Day et al., 2004). Furthermore, in dissociating the neurocircuitry for TMT-induced fear from the circuitry for other predator odors, a comprehensive excitotoxic and electrolytic lesion study of the three nuclei of the medial hypothalamic defense circuit found that excitotoxic lesions of any of the three nuclei did not reduce TMT-induced freezing (Pagani and Rosen, 2009). However, the same study showed that electrolytic lesions in the anterior hypothalamic nucleus and VMHdm significantly reduced TMT-induced freezing. Interestingly, these electrolytic lesions reduced shock-induced contextual fear conditioning, identifying another dissociation of innate fear and learned fear neural circuitry.

Given that electrolytic lesions, but not excitotoxic lesions, of the VMHdm and anterior hypothalamic nucleus reduced TMT-induced freezing, the results suggest that axonal fibers passing through these regions are likely part of a circuit for TMT-induced freezing. One possible candidate is a pathway from the bed nucleus of the stria terminalis (BNST) to the PAG (Dong and Swanson, 2004, 2005, 2006), which passes through the anterior hypothalamic nucleus and VMHdm (Figure 1F). Inactivation of the ventral BNST with muscimol or norepinephrine antagonism with clonidine has been shown to block TMT-induced freezing (Fendt et al., 2003, 2005b; Fendt and Endres, 2008). Furthermore, TMT induces immediate-early gene activation in the medial and lateral aspects of the BNST (Day et al., 2004; Asok et al., 2013). Immediate-early gene expression is also blocked in DII olfactory sensory neuron knockout mice exposed to TMT (Kobayakawa et al., 2007). Because the medial nucleus of the amygdala innervates the medial parts of the BNST, a putative pathway for TMT-induced freezing could be zone II of the nasal epithelium or Grueneberg ganglion—dorsal main olfactory bulb—cortical amygdala—medial amygdala—medial bed nucleus of the stria terminalis—ventrolateral periaqueductal gray (Figure 1A). However, an alternative pathway might be through the lateral septum since inactivation of the lateral septum has been shown to block TMT-induced freezing (Endres and Fendt, 2008). The lateral septum has reciprocal connections with the amygdala, bed nucleus of the stria terminalis, and periaqueductal gray to be incorporated into the circuit described above. An interesting article on social aggregation of rats as a defense against prey, demonstrated that rats with a more active response to cat fur odor displayed less c-fos activation in the lateral septum suggesting that the septum may be involved in defensive aggregation (Bowen et al., 2013).

## TMT-induced fear: Neuroanatomical pathways compared to the fear conditioning neuroanatomical pathway

A putative, partial neuroanatomical circuit for TMT-induced innate fear behavior is laid out in Figure 1A. The neuroanatomical circuit for fear conditioning and fear-conditioned behavior is well delineated (Figure 1B) and will not be detailed here (see Ledoux, 2000; Davis and Whalen, 2001; Davis et al., 2010; Dejean et al., 2015, for detailed descriptions of the circuit). What is obvious about the TMT circuit is that it differs from the canonical circuit for fear conditioning and fear conditioned defensive behavior (freezing, startle), which contains the basolateral and central amygdala complexes. The basolateral complex (which contains the lateral and basal nuclei of the amygdala) and the central complex (which contains the lateral and medial divisions of the central nucleus of the amygdala) have been shown to be central and crucial for foot-shock induced fear conditioning with various conditioned stimuli including sounds, lights, contexts, and smells. Because TMT-induced freezing is not learned, the pathway appears to bypass the basolateral and central complexes, and instead looks as if it proceeds from the olfactory bulb through the amygdala cortex, medial amygdala nucleus, and medial bed nucleus of the stria terminalis to the periaqueductal gray (See previous section). Lesions or inactivation of the basolateral or central nuclei of the amygdala produce only small reductions or only a delay in TMT induced-freezing (Wallace and Rosen, 2001; Fendt et al., 2003; Rosen, 2004; Müller and Fendt, 2006), indicating these amygdala nuclei so important for fear conditioning, are not necessary for TMT-induced freezing, but seem to modulate TMT effects. Corroborating the minimal lesion and inactivation effects, immediate-early gene (c-fos, egr-1) expression is not increased following TMT exposure in the basolateral complex of the amygdala in rats (Day et al., 2004; Staples et al., 2008; Asok et al., 2013), but see Hebb et al. (2004) in mice.

The lack, or minimal effects, of inactivation and lesions of the basolateral and central complexes of the amygdala on TMT-induced freezing does not suggest that these regions are not involved in olfactory-motivated behavior. In fact, a number of studies demonstrate that the basolateral complex of the amygdala is important for olfactory fear conditioning, where once a non-predator odor is paired with a footshock, it elicits fear responses (Cousens and Otto, 1998; Sevelinges, 2004; Walker et al., 2005; Jones et al., 2007; Hegoburu et al., 2014). It appears likely that the basolateral complex is critical for olfactory-fear conditioning as it is for auditory and visual fear conditioning induced by footshock, but not for innate TMT-induced freezing. In contrast, as discussed above, the basolateral complex may only modulate TMT-induced freezing by delaying the full expression of freezing or slightly reducing the amount of freezing (Wallace and Rosen, 2001; Fendt et al., 2003).

The role of the central nucleus of the amygdala in olfactory fear conditioning is less certain because there are very few studies in which this nucleus is inhibited or lesioned. One study in infant rats showed that lesions of the central nucleus interfered with olfactory fear conditioning with shock (Sananes and Campbell, 1989). However, c-fos expression in the central nucleus of the amygdala was not increased in olfactory fear conditioning in adult rats (Schettino and Otto, 2001), nor was c-fos increased in the central nucleus of the amygdala with context conditioning induced by cat fur odor (Dielenberg et al., 2001; Staples et al., 2005). This contrasts with the very large increase in c-fos, egr-1, CART, and CRH in the central nucleus of the amygdala with exposure to TMT (Day et al., 2004; Staples et al., 2008; Asok et al., 2013; Sharma et al., 2014).

Another region of the extended amygdala—the bed nucleus of the stria terminalis—is not only important in TMT-induced freezing, but is also involved in sustained fear (Davis et al., 2010). Sustained fear would include contextual fear conditioning (Sullivan et al., 2004), fear conditioning with prolonged conditioned fear stimuli (Waddell et al., 2006; Davis et al., 2010), fear sensitization (Davis and Walker, 2014), fear generalization (Duvarci et al., 2009), and also innate fear of TMT (Fendt et al., 2003). Interestingly, if the basolateral amygdala complex is inactivated during contextual fear conditioning, the BNST can take over the function of the basolateral amygdala complex (Ponnusamy et al., 2007; Poulos et al., 2010; Zimmerman and Maren, 2011). Thus, the BNST is involved in more than just innate fear of predator odor, but has a functionally more rich involvement in fear and anxiety.

## What we know about the neuroanatomy of TMT-induced fear behavior

Research on the neuroanatomy of TMT-induced fear has progressed to where there appears to be a specialized circuit from the nasal epithelium to the main olfactory bulb to the cortical amygdala. From there we don't quite know the circuit, but from the cortical amygdala it possibly continues to the medial amygdala nucleus, then bed nucleus of the stria terminalis, before finally reaching the periaqueductal gray for freezing. The lateral septum is also likely part of this pathway, but how it is connected is not known. We also know that the prelimbic cortex also modulates TMT-induced freezing, but inactivation enhances freezing, suggesting it plays an inhibitory role (Fitzpatrick et al., 2011).

Importantly, the basolateral and central complexes of the amygdala, so essential for fear conditioning, do not seem to play primary roles in TMT-induced fear. The data summarized above suggest there is a divergence of pathways for conditioned fear and innate fear. The dual neural pathways for learning about environmental contingencies of olfactory stimuli tracking through the basolateral and central complexes of the amygdala on the one hand, and hardwired pathways for innate sensory coding of ecologically significant odors on the other, is also evident in simpler animals than mammals (Vosshall and Stocker, 2007). For example, drosophila has two olfactory pathways, one through the antenna lobe (analogous to the olfactory bulbs) to the mushroom bodies, a region involved in olfactory learning and memory (possibly analogous to the pyriform cortex in mammals), and the lateral horn, a region mediating direct behavioral responses to odors (possibly analogous to the cortical amygdala) (Vosshall and Stocker, 2007). This type of divergent neural circuitry for learning vs. innate coding for threat (and appetitive odors, Root et al., 2014) seems to be a common motif in the animal kingdom.

## What we still need to learn about the neuroanatomy of TMT-induced fear behavior

Although the olfactory circuit through the cortical amygdala is fairly well delineated, the actual olfactory receptors responsible for TMT's innate properties are not well defined. The Domain II (DII) population of olfactory receptors comprises about 100 olfactory receptor genes (Kobayakawa et al., 2007), but which specific ones are responsible for TMT-induced freezing are not known. The identification of the specific olfactory receptor(s) that bind TMT and transduce TMT's innate fear properties, similar to what has been accomplished for olfactory receptors for 2-phenylethylamine (the TAAR4 receptor, Ferrero et al., 2011) and for Feld4 from cat fur/saliva and rMUP13 from rat urine (the TrpC2 receptor, Papes et al., 2010), would help propel the neuroanatomical circuitry research on TMT further.

Interestingly, there are also olfactory receptors in more ventral regions of the olfactory bulb. TMT can be used as a discriminative stimulus for positive reward in mutant mice devoid of the dorsal Domain II glomeruli, apparently through the TMT-responsive receptors in the ventral olfactory bulb (Kobayakawa et al., 2007). Possibly, these ventrally located neurons project to the pyriform cortex, which is activated by TMT (Illig and Haberly, 2003), as opposed to the cortical amygdala, to transmit TMT-induced olfactory information for involvement in olfactory learning (Kobayakawa et al., 2007; Miyamichi et al., 2010; Root et al., 2014).

An additional brain region that receives olfactory input from the main olfactory bulb is the entorhinal cortex (Insausti et al., 2002), which has major projections to the hippocampus. The hippocampus is particularly involved in contextual fear conditioning induced by coyote urine (Wang et al., 2012, 2013).

Another question also remains—how are the various regions of the circuit wired to each other? The part of the circuit from the nasal epithelium to cortical amygdala is pretty well characterized, but from there it is unclear how the circuit progresses to the PAG to elicit freezing behavior. Are projections from the cortical amygdala to the medial nucleus of the amygdala important? Is a direct pathway from the cortical amygdala to the bed nucleus of the stria terminalis involved? Or is the pathway from the medial nucleus of the amygdala to the ventral and medial divisions of the bed nucleus of the stria terminalis necessary? Does a direct pathway from the BNST to the PAG convey the TMT-induced fear signal for freezing?

Further delineation of the parts of the BNST that are involved in the various types of fear need to be addressed. The ventral and medial nuclei of the BNST are important for TMT-induced fear (Fendt et al., 2003; Müller and Fendt, 2006). However, the dorsolateral division of the BNST (also called the oval nucleus of the BNST) shows high levels of immediate-early gene expression following TMT exposure (Day et al., 2004; Asok et al., 2013). This is the region of the BNST that is implicated in sustaining learned fear and anxiety (Davis et al., 2010). How the dorsal and ventral divisions of the BNST interact for TMT-induced fear would be of particular interest for understanding the circuitry and modulation of innate predator fear and other types of fear.

There may also be other circuits involved in TMT-induced behaviors that route through areas outside of the BNST. Inactivation of the lateral septum significantly reduces freezing to TMT, but how it interacts with the proposed circuit (Figure 1) is not clear. The lateral septum strongly projects to the anterior nucleus of the hypothalamus, which is part of the cat and cat fur induced defensive circuit (Gross and Canteras, 2012), but the anterior nucleus of the hypothalamus does not seem to be part of the circuit for TMT-induced freezing (Pagani and Rosen, 2009). Furthermore, a number of neurotransmitter system modulate TMT-induced freezing, but we have not learned much beyond the initial studies demonstrating an effect. For example, norepinephrine infused into the ventral BNST enhanced TMT-induced freezing, while an α_2_-antagonist blocks this norepinephrine enhancement and blocked TMT-induced freezing on its own (Fendt et al., 2005b). Chemical lesions of acetylcholine producing neurons also reduce TMT-induced freezing (Knox et al., 2008). However, more studies on the modulatory role of various neurotransmitters will be informative about autonomic and attentional mechanism of innate fear.

What we know and still need to learn about the anatomy of TMT-induced fear is summarized in Table 2.

**Table 2:** **Neuroanatomy of TMT-induced fear**.

What we know about the neuroanatomy of TMT-induced fear A pathway from olfactory neurons in the nasal epithelium and Grueneberg ganglion to the amygdala cortex has been delineated - Zone II receptors in nasal epithelium process innate fear to TMT - Genetic deletion of zone II olfactory sensory neurons eliminate fear responses to TMT - These olfactory sensory neurons synapse on mitral cells project to the amygdala cortex - Optogenetic inactivation of this projection to the amygdala cortex blocks TMT-induced freezing Olfaction, but not nociception, of TMT is critical for TMT-induce freezing Medial nucleus of the amygdala and bed nucleus of the stria terminalis are part of a proposed circuit for TMT-induced fear - Inactivation of these regions block TMT-induced freezing The medial hypothalamic defensive circuit is not critical for TMT-induced freezing - Fiber-sparing lesions of nuclei of the medial hypothalamic defensive circuit do not block TMT-induced freezing, but lesions that destroy fibers passing through this circuit block TMT-induced freezing
What we still need to learn about the neuroanatomy of TMT-induced fear What are the specific olfactory receptors for TMT-induced fear? - About 100 receptors are possible, further refinement is important What is the role of the pyriform and entorhinal cortex in TMT-induced fear and fear conditioning? How are the various regions of the circuit wired? - Projects from the amygdala cortex to the medial nucleus of the amygdala and bed nucleus of the stria terminalis are ill defined. - Is a direct project from the bed nucleus of the stria terminalis to the periaqueductal gray part of the circuit? Or is there an indirect pathway? - How are other regions that affect TMT-induced fear (e.g., lateral septum, prefrontal cortex) wired into the circuit? - How many circuits for TMT-induced fear are there? - How similar and divergent are circuits for fear to various predator odors?

## Conclusions

Great progress has been made in the last two decades on understanding the neural basis of both learned fear and innate fear. Detailed progression on neural circuitries from olfaction to defensive behavior is within reach. Identification of single molecules that elicit defensive and fear responses allows for precise determination of the olfactory receptor, or receptors, involved in the transduction of the olfactory threat signals. Investigation using TMT as a threatening stimulus for rodents has been particularly important in this advancement. Behavioral studies have demonstrated the robustness of TMT-induced freezing, which has allowed for the use of traditional lesion/inactivation manipulations and modern optogenetic and molecular techniques to accurately localize olfactory neurons and the functional neural pathways responsible for perception of predator odor threat and the generation of defensive behavior. Comparison of the neural circuitry for TMT with other predator odors is critical for delineating the common and unique circuits for predator odors. This would not only be at the olfactory receptor stage, but at regions and synapses throughout the neural circuits, where differences between TMT and cat-derived odors are already evident (Pagani and Rosen, 2009; Gross and Canteras, 2012; Pérez-Gómez et al., 2015).

Finally, continued behavioral investigations on the generation of TMT-induced behaviors other than freezing will be important for broadening the scope of analysis into multiple circuitries for various species selective defensive responses. Further, examination of the innate properties of TMT-induced behavior and the constraints of fear learning supported by TMT and other predator odors will be important for understanding innate and learned fears and phobias (Rosen et al., 2008b), and then possible treatments.

## Author contributions

JR, AA, and TC all contributed to the conception, drafting, and revising of the work critically for important intellectual content. JR, AA, and TC gave their final approval of the version to be published and agree to be accountable for all aspects of the work in ensuring that questions related to the accuracy or integrity of any part of the work are appropriately investigated and resolved.

## Funding

The work was partially funded by grants P20GM103653 and R01HD075066 from the National Institutes of Health.

### Conflict of interest statement

The authors declare that the research was conducted in the absence of any commercial or financial relationships that could be construed as a potential conflict of interest.
